# A sentiment analysis approach to the prediction of market volatility

**DOI:** 10.3389/frai.2022.836809

**Published:** 2022-12-20

**Authors:** Justina Deveikyte, Helyette Geman, Carlo Piccari, Alessandro Provetti

**Affiliations:** ^1^Department of Computer Science, Birkbeck, University of London, London, United Kingdom; ^2^Department of Economics, Mathematics and Statistics, Birkbeck, University of London, London, United Kingdom

**Keywords:** sentiment analysis, Twitter, news analytics, volatility, topic modeling (LDA)

## Abstract

Prediction and quantification of future volatility and returns play an important role in financial modeling, both in portfolio optimisation and risk management. Natural language processing today allows one to process news and social media comments to detect signals of investors' confidence. We have explored the relationship between sentiment extracted from financial news and tweets and FTSE100 movements. We investigated the strength of the correlation between sentiment measures on a given day and market volatility and returns observed the next day. We found that there is evidence of correlation between sentiment and stock market movements. Moreover, the sentiment captured from news headlines could be used as a signal to predict market returns; we also found that the same does not apply for volatility. However, for the sentiment found in Twitter comments we obtained, in a surprising finding, a correlation coefficient of –0.7 (*p* < 0.05), which indicates a strong negative correlation between negative sentiment captured from the tweets on a given day and the volatility observed the next day. It is important to keep in mind that stock volatility rises greatly when the market collapses but not symmetrically so when it goes up (the so-called leverage effect). We developed an accurate classifier for the prediction of market volatility in response to the arrival of new information by deploying topic modeling, based on Latent Dirichlet Allocation, in order to extract feature vectors from a collection of tweets and financial news. The obtained features were used as additional input to the classifier. Thanks to the combination of sentiment and topic modeling even on modest (essentially personal) architecture our classifier achieved a directional prediction accuracy for volatility of 63%.

## 1. Introduction

Reliably predicting stock market returns and volatility (the degree of variation of a trading price series over time) has attracted much attention from academics as well as the financial industry for more than 60 years now with the founding work of Nobel laureates Markowitz, Sharpe, Lintner, and many others after them. But can stock market prices and volatility be predicted? Or at least, can they be predicted at some specific time? We address such general question from a very specific angle: can contemporary techniques for Online social media analysis find signals that lead to prediction, and can such analysis be deployed on modest architectures, i.e., essentially, as a personal computing project?

Measuring sentiment captured from online sources such as Twitter or financial news articles can be valuable in the development of trading strategies. In addition, sentiment captured from financial news can have some predictive power that can be harnessed by portfolio and risk managers.

Figure 1 is an overview of our experimental architecture; it has been implemented on PCs and can be ran daily. We have instrumented three sources, which will be defined in detail later: Twitter messages, Headlines and ‘news stories' appearing online on financial channels. The results and conclusions from our analysis can be classified into three parts. First, correlations between sentiment scores and stock market returns were statistically significant for our headline dataset only (neither Twitter nor New stories provided valuable signals). Second, a statistically-significant negative correlation between negative news and the closing price of FTSE100 index (returns). Third, the strongest correlation between sentiment and volatility measures was detected in our tweets dataset, while no correlation or weak correlation was found in headlines and news stories dataset. We anticipate our conclusions by observing that the observed behaviors can be explained by the fact that tweets are normally timelier and more reactive to various events, whereas it takes much more time to publish articles, and the market functions according to the principle “buy on rumors, sell on news.”

**Figure 1:**
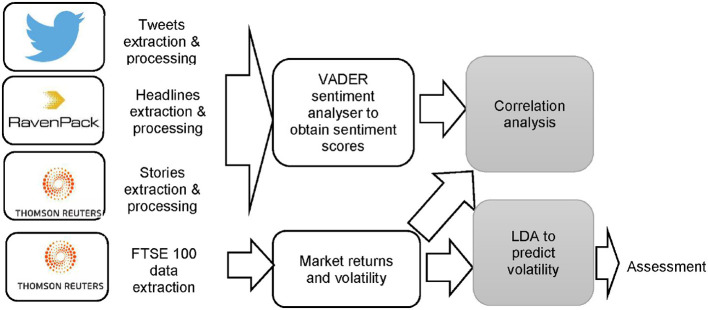
Overview of our software architecture and dataflow.

This article is structured as follows. In Section 2 we review related research. In Section 3, we describe data sources which have been used to calculate sentiment scores: Thomson Reuters, RavenPack, and Twitter. In Section 3 we conduct a correlation analysis and Granger's causality test. In Section 3.4, we carry out additional experiments to investigate whether topic modeling or Latent Dirichlet Allocation can be used to enhance the prediction accuracy of the next-day stock market directional volatility. In Sections 4, 5 we present the results of the analysis and discuss future work.

## 2. Related works

A growing number of research papers use Natural Language Processing (NLP) methods to analyze how sentiment of firm-specific news, financial reports, or social media impact stock market returns. An important early work by Tetlock (2007) explores possible correlations between the media and the stock market using information from the Wall Street Journal and finds that high pessimism causes downward pressure on market prices. A year later, Tetlock et al. (2008) deployed a *bag-of-words* model to assess whether company financial news can predict a company's accounting earnings and stock returns. The results indicate that negative words in company-specific news predict low firm earnings, although market prices tend to under-react to the information entrenched in negative words.

Bollen et al. (2011) examined whether sentiment captured from Twitter feeds is correlated with the value of the Dow Jones Industrial Average Index (DJIA). They deployed OpinionFinder and Google's Profile of Mode State (GPOMS), two opinion-tracking tools that measure mood in six dimensions (Calm, Alert, Sure, Vital, Kind, and Happy). The obtained measures led them to conclude, in their words, that “the accuracy of DJIA predictions can be significantly improved by the inclusion of specific public-mood dimensions.”

At about the same time (Loughran and McDonald, 2011) applied sentiment analysis to the so-called 10-K filings. They found that almost three-quarters of negative word counts in 10-K filings based on the Harvard dictionary are typically not negative in a financial context. To do so, they developed an alternative dictionary that better reflects sentiment in a financial text.

Mao et al. (2011) used a wide range of news data and sentiment tracking measures to predict financial market values. The authors find that Twitter sentiment is a significant predictor of daily market returns, but after controlling for all other mood indicators including VIX, sentiment indicators are no longer statistically insignificant. Recently, Calomiris and Mamaysky (2018) used news articles to develop a methodology to predict risk and return in stock markets in developed and emerging countries. Their results indicate that the topic-specific sentiment, frequency and “surprise” of news text can predict future returns, volatility, and *drawdowns*.

### 2.1. Works on volatility

Whereas, a majority of the literature works in text mining/sentiment analysis seem to focus on predicting market prices or directional changes only few works looked into how financial news impacts stock market volatility. One of them is Kogan et al. (2009) which used Support Vector Machine (SVM) to predict the volatility of stock market returns. Their results indicate that text regression corelates well with current and historical volatility and a combined model performs even better. Similarly, Hautsch and Groß-Klußmann (2011) found that the release of highly relevant news induces an increase in return volatility, with negative news having a greater impact than positive news.

Glasserman and Mamaysky (2019) used an N-gram model, which they applied to as many as 367,311 articles, to develop a methodology showing that unusual negative and positive news forecasts volatility at both the company-specific and aggregate levels. The authors find that an increase in the “unusualness” of news with negative sentiment predicts an increase in stock market volatility. Similarly, unusual positive news forecasts lower volatility. According to their findings, news are reflected in volatility more slowly at the aggregate than at the company-specific level, in agreement with the effect of diversification. The somehow-parallel approach by Caporin and Poli (2017) also found that news-related variables can improve volatility prediction. Certain news topics such earning announcements and upgrades/downgrades are more relevant than other news variables in predicting market volatility.

In a more recent study, Atkins et al. (2018) used LDA and a simple Naive Bayes classifier to predict stock market volatility movements. The authors found that the information captured from news articles can predict market volatility more accurately than the direction the price movements. They obtained a 56% accuracy in predicting directional stock market volatility on the arrival of new information. The authors concluded that “volatility movements are more predictable than asset price movements when using financial news as machine learning input, and hence could potentially be exploited in pricing derivatives contracts *via* quantifying volatility.” Apart from volatility, another deployment of LDA is due to Mahajan et al. (2008) who used it to identify topics of financial news and then to predict a rise or fall in the stock markets based on topics extracted from those news. Their classifier achieved 60% accuracy in predicting market direction.

Jiao and Walther (2016) show that a high social media activity around a specific company predicts a significant increase in return volatility whereas attention from influential press outlets, e.g., the Wall Street Journal in fact is a predictor of the opposite: a decrease in return volatility. Finally, we can only mention early works on connecting text, e.g., disclosure reports and business news to volatility have appeared in non-AI journals. For instance, Kothari et al. (2009) related an extensive list of corporate disclosures to return volatility. Their main finding was that positive news disclosures lower volatility, while negative disclosures increase volatility.

## 3. Data and methods

We focus on the FTSE100 index as it is commonly understood to represent the best indicator of the health of the UK stock market: it reflects the performance of the largest 100 companies listed on the London Stock Exchange (LSE) with the highest market capitalization. The daily closing prices of FTSE100 index were again obtained from Reuters' Eikon Platform simply using their API. In order to assess the relationship between stock market movements and sentiment we computed daily market returns and defined the return on day *t* as the log-change of Close from day *t*-1, which is expressed as follows:


(1) $$\begin{array}{l} r_{t}=\log\frac{CLOSE_{t}}{CLOSE_{t-1}} \end{array}$$

The volatility of FTSE 100 is classically defined as:


(2) $$\begin{array}{l} Vol=\sqrt{\frac{1}{N}\overset{N}{\underset{t+1}{\sum }}{\left(r_{t}-\bar{r}\right)}^{2}}\cdot \sqrt{252} \end{array}$$

For our research we chose to use three different data sets (tweets, news headlines about FTSE100 companies, and full news stories) to analyze sentiment and compare the results. News headlines about FTSE100 companies were obtained from RavenPack. The dataset includes headlines as well as other metadata collected from January to August 2019. News arrival is recorded with GMT time stamps up to a millisecond precision. In total we have 969,753 headlines for our analysis. The number of headlines during the weekends ranged from around 700 to 1,300 daily, while during normal working days the number of headlines often exceeded 5,000 per day. Thanks to the Eikon API ^1^ we were able to gather news stories about FTSE100 companies. Around 12,000 articles have been collected between April and August 2019. Meanwhile, by using Twitter Streaming API, we collected a total of 545,979 tweets during the months of July and August 2019. For the purpose of this study and in order to avoid too generic tweets, we retained and mined only the so-called “$cashtags” that mentioned companies included in the FTSE100 index. The rationale for selecting certain hashtags relates back to the original aim of measuring sentiment of news related to FTSE100 companies rather than the overall financial industry.

### 3.1. The VADER sentiment intensity analyzer

VADER is a lexicon and rule-based sentiment analysis tool that is tuned to capture sentiments expressed in social media. This tool was first developed by C.J. Hutto and E.E. Gilbert in 2014, but since then it underwent several improvements and updates. The VADER sentiment analyzer is extremely accurate when it comes to social media texts because it provides not only positive/negative scores but also a numeric measure of the intensity of the sentiment. Another advantage of using VADER is that it does not need training data as it uses human labeled sentiment lexicon and works fairly fast even on simple laptops.

The VADER lexicon was created manually^2^; ten independent human raters annotated more than 9,000 token features on the following –4 to +4 scale:

- Extremely negative: –4,
- Neutral score: 0, and
- Extremely positive: +4.

The positive, negative, and neutral scores are ratios for the proportions of text that fall in each category and should sum to 1. The compound score is derived by summing the sentiment scores of each word in the lexicon, adjusted according to the rules, and then normalized to be between –1 (most extreme negative) and +1 (most extreme positive). This is the most useful metric if we want a single uni-dimensional measure of sentiment for a given sentence.

Another feature that made VADER the right tool for our experiments is that its sentiment analyzer can handle negations and UTF-8-encoded emojis, as well as acronyms, slang and punctuation. Furthermore, it takes punctuation into account by amplifying the sentiment score of the sentence proportionally to the number of exclamation points and question marks ending the sentence. VADER first computes the sentiment score of the sentence. If the score is positive then VADER adds a certain empirically-obtained score for every exclamation point (0.292) and question mark (0.18). Conversely, negative scores are subtracted.

### 3.2. Aggregating news and sentiment scores

In contrast to financial stock data, news and tweets were available for each day, although the number of tweets and news was significantly lower during weekends and bank holidays. Not to waste such information, we decided to transfer the sentiment scores accumulated for non-trading days to the next nearest trading day. That is, the average news sentiment prevailing over weekend will be applied to the following Monday. The same logic holds for holidays.

For our daily analysis, we aggregate sentiment scores captured from all tweets on day *t* to access its impact on the stock market performance in the coming *t*+1 day. For instance, we aggregate sentiment captured from tweets on July 10 to analyze the correlation between sentiment on the 10th/11th July and market volatility and returns.

We have adopted the following (and fairly classical) definition of the daily, or compound, sentiment score, *Sent*_*d*_, from Gabrovsek et al. (2016):


(3) $$\begin{array}{l} Sent_{d}=\frac{N_{d}\left(pos\right)-N_{d}\left(neg\right)}{N_{d}\left(pos\right)+N_{d}\left(neut\right)+N_{d}\left(neg\right)+3} \end{array}$$

where *Nd(neg), Nd(neut)*, and *Nd(pos)* denote the daily volume of negative, neutral, and positive tweets. The sentiment score is thus the mean of a discrete probability distribution and, as Gabrovsek et al. (2016) put it, has “values of –1, 0, and +1 for negative, neutral and positive sentiment, respectively. The probabilities of each label are estimated from their relative frequencies, but when dealing with small samples (e.g., only a few tweets about a stock per day) it is recommended to estimate probabilities with Laplace estimate.” Table 1 shows, on a few selected days, the values we obtained from VADER.

**Table 1:** Twitter dataset: The aggregated (compound) sentiment scores computed with VADER.

**Date created**	**Negative**	**Positive**	**Neutral**	**Compound**
2019-08-02	0.087	0.224	0.690	0.209
2019-08-03	0.082	0.250	0.668	0.238
2019-08-04	0.087	0.248	0.666	0.232
2019-08-05	0.103	0.236	0.660	0.192
2019-08-06	0.084	0.246	0.670	0.235

### 3.3. Causality testing

To verify whether market sentiment can indeed be useful for predicting FTSE100 Index movements, we decided to perform a Granger's causality test. Such method is used to identify causality between two variables and whether one time series variable could be important in forecasting the other. In our case, we test whether the sentiment obtained from financial news and social media (variable *X*) could be useful in forecasting the stock market performance and volatility (variable *Y*):


(4) $$\begin{array}{l} y_{t}=\alpha +\overset{k}{\underset{i=1}{\sum }}\beta_{j}Y_{t-1}+\overset{k}{\underset{j}{\sum }}\lambda_{j}X_{t-j}+\epsilon_{t} \end{array}$$

If the *p*-value is less than 0.05, we could reject the null hypothesis and conclude that variable *X* (sentiment) influences stock market changes and volatility. Granger's test provides insights into how much predictive information one signal has about another one over a given lagged period. Here the *p*-value measures the statistical significance of the causality between two variables (sentiment and market returns).

### 3.4. Topic discovery

The most commonly-used method for topic modeling, or topic discovery from a large number of documents, is Latent Dirichlet allocation (LDA). LDA is a generative topic model which generates combination of latent topics from a collection of documents, where each combination of topics produces words from the collection's vocabulary with certain probabilities. The process of running LDA analysis consists of several steps. A distribution on topics is first sampled from a Dirichlet distribution, and a topic is further chosen based on this distribution. Moreover, each document is modeled as a distribution over topics, and a topic is represented as a distribution over words.

LDA allows a set of news stories and tweets to be categorized into their underlying topics. According to Atkins et al. (2018) “a topic is a set of words, where each word has a probability of appearance in documents labeled with the topic. Each document is a mixture of corpus-wide topics, and each word is drawn from one of these topics. From a high-level, topic modeling extrapolates backwards from a set of documents to infer the topics that could have generated them—hence the generative model.” Although LDA reduces the dimensionality of the data by producing a small number of topics, it is relatively heavy computationally as its cost grows with *O*(*n*^*k*^). We have followed Atkins' methodology to assess whether topics extracted from tweets and news headlines can be used to predict directional changes in market volatility. Let us now describe the steps we took to perform LDA and use the obtained topic distribution to predict next day's market volatility (“UP” or “DOWN”).

First, we followed Kelechava's methodology^3^ to convert topics into feature vectors. Then, an LDA model was used to get the distribution of 15 topics for every day's headlines. This 15-dimensional vector will be used later as a feature vector for a classification problem, to assess whether topics obtained on a certain day can be used to predict the direction of market volatility the next day.

The feature vector for an interval is a topic-count sparse vector, it represents the number of times each topic appears in headlines/tweets or articles within the given interval. Some topics may appear more than once, and some not at all. The target vector is then constructed by pairing binary direction labels from market volatility data to each feature vector. For instance, we are using headlines from day *t* to predict the direction of movement (increase/decrease) of volatility the next day.

## 4. Results

Financial markets are influenced by a number of quantitative factors, ranging from company announcements and performance indicators such as EBITDA, to sentiment captured from social media and financial news. As described in Section 2, several studies have modeled and tested the association between “signals,” i.e., sentiment, from the news and market performance. To evaluate our own sentiment extraction we have applied Pearson's correlation coefficient to quantify the level of correlation between sentiment of our data collection, which was presented by example in Table 1, and stock market volatility and returns.

### 4.1. Sentiment from the headlines

The analysis of our headlines collection confirmed the expectation of a negative correlation between negative sentiment and stock market returns for a given day t. The results of the analysis are available from Figure 2:

**Figure 2:**
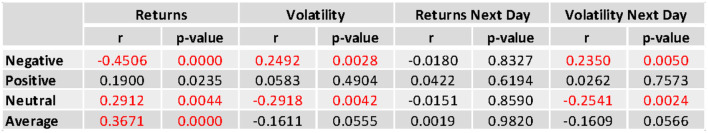
Headlines dataset: Correlation coefficients and *p*-values for lagged and non-lagged returns; statistically-relevant results are in red.

The correlation coefficient r is equal to –0.45 and the *p*-value, in Figure 2 is below 0.05 so we can reject the null hypothesis and conclude that the relationship between negative sentiment captured from the headlines is moderate and statistically significant.

We can also interpret this correlation as follows: if the sentiment of the headlines becomes increasingly negative then the closing price of the FTSE 100 index would decrease. In other words, when negative sentiment increases, returns decline and vice versa. The average sentiment score was obtained by using formula 3 and the highest *Sent*_*d*_ value is, the stronger positive sentiment is and vice versa. We obtained an r of 0.37 which indicates a weak correlation between average sentiment and stock market returns. Also, if the average sentiment score increases the stock market returns will increase too, but if the average score decreases (becomes negative) then stock market returns would decrease as well. Figure 3 illustrates this relationship.

**Figure 3:**
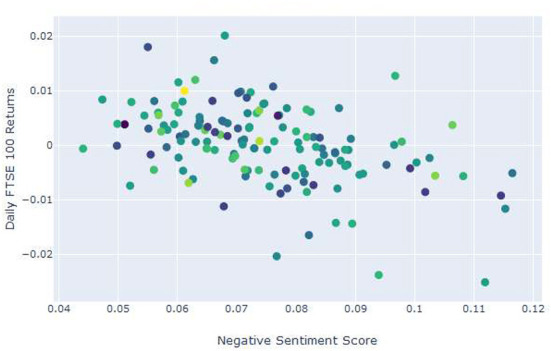
Headlines dataset: The scatterplot shows the linear relationship between negative sentiment and returns.

The next test was on delayed influence, i.e., returns the next day. To evaluate time-lag correlations between sentiment (again, from the headlines) and stock market returns we computed cross-correlation using a time lag of 1 day. The results indicate that there is no statistically significant correlation between sentiment scores and market returns next day. However, there is weak positive correlation between negative sentiment at day t and the volatility of the next day. *R*-value of 0.24 and *p*-value below 0.05 indicate that the two variables (negative sentiment and volatility) move in tandem. For instance, if the negative sentiment at a given day *t* increases, the volatility of the market would also increase the next day.

### 4.2. Sentiment from the tweets

We also tested the association between sentiment captured from tweets and stock market returns and volatility. The type of values we were getting from the VADER analysis of our tweets are shown in Table 1.

For Twitter, our findings, in Figure 4, are somewhat similar to the literature but for a strong correlation between sentiment (positive, neutral and average) and stock market volatility. In particular, a correlation coefficient of –0.7, and a *p*-value below 0.05 indicate that there is a strong negative correlation between positive sentiment and the volatility of the market next day. This finding can be interpreted as follows: as the positive sentiment increases, market volatility decreases (variables move in opposite directions).

**Figure 4:**
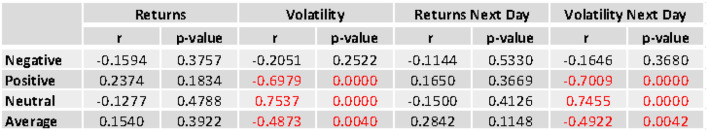
Tweets dataset: Correlation coefficients and *p*-values for lagged and non-lagged returns; statistically-relevant results are in red.

### 4.3. Sentiment from news stories

The results obtained from the correlation analysis using new stories datasets show a weak correlation between sentiment and FTSE 100 returns and returns. However, none of the correlation values were statistically significant. This lack of signal could however be explained by the limitations of our dataset: after cleaning and pre-processing we only had around 10,000 full news stories left.

### 4.4. Summary of findings about sentiment

The results above from thee different data sets suggest that the relationship between market sentiments and stock prices can be quite complex and may exist only in some of the time periods. It is unsurprising that the market exhibited different behaviors in different time periods. Overall, our results confirm the expectation of positive correlation between average sentiment scores in relation to returns of the stock market and a negative correlation between positive sentiment and the volatility of stock market (as the average sentiment increases toward more positive the market returns tend to increase as well, and the opposite happens with volatility: more positive news mean calmer markets and less volatility). As the correlation coefficients (-0.45, 0.29 and 0.37) are significantly below 0.5, it can be concluded that the relationship between the sentiment (negative, neutral, average) and the market returns at a given day t is relatively weak but statistically significant. This means that sentiment captured from headlines can be potentially used as a signal to predict the closing price of our FTSE 100 index. For next day returns, the correlation coefficients are very low and statistically insignificant in all three datasets. This means that our current sentiment analysis architecture cannot reveal signals that help predicting market returns the next day. Yet the opposite can be said about correlation between sentiment and market volatility.

### 4.5. Results on causality testing

Our causality testing exhibited no reliable causality between the sentiment scores and the FTSE100 return with any lags. We found that causality slightly increased at a time lag of 2 days but it remained statistically insignificant. Vice versa Granger's text found statistical significance in negative returns causing negative sentiment, as expected. To summarize, the Granger's causality analysis of three different datasets (Headlines, News stories and Tweets) against FTSE returns and volatility has shown that, in general, sentiment obtained from news or social media was found to “cause” neither changes to the FTSE100 index closing prices nor changes in market volatility. The *p*-values were all above the significance threshold, which means our null hypothesis could not be rejected.

### 4.6. Applying the classifier to unseen test sets

Thanks to the preparation described earlier, we could build a dedicated LDA model and train our classifier. We tested our model by computing a feature vectors from unseen test data and running a simple logistic regression model to predict whether the next day's market volatility will increase or decrease, as in Figure 5.

**Figure 5:**
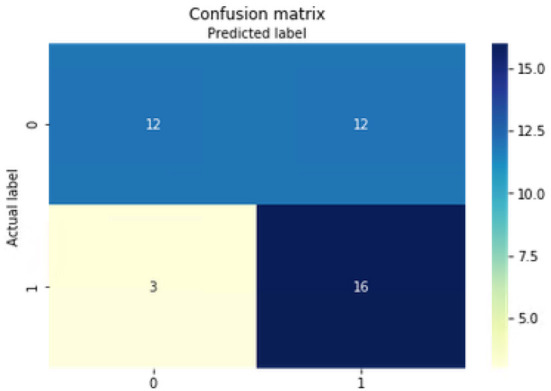
Confusion matrix for our headlines dataset.

Our model applied to the headlines dataset obtained an accuracy of 65%. This indicates that topics extracted from news could be used as a signal to predict the direction of market volatility next day. The results obtained from our experiment are similar to those of Atkins et al. (2018) and Mahajan et al. (2008). The accuracy was slightly lower for the tweets dataset, which can be explained by the fact that tweets text typically contains abbreviations, emojis and grammatical errors which could make it harder to capture topics from tweets.

### 4.7. Visualizing the emergent topics

The “tag cloud” presented in Figure 6 was obtained from headlines dataset. Each topic contains a maximum of 10 words. It is interesting to notice that topics captured from headlines news are very different from those obtained from the news stories.

**Figure 6:**
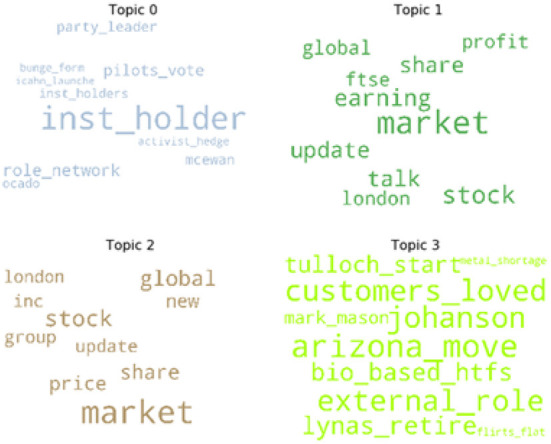
IDA topic modeling of our headlines corpus.

In Figure 7 we see the main topics extracted from the News stories dataset.

**Figure 7:**
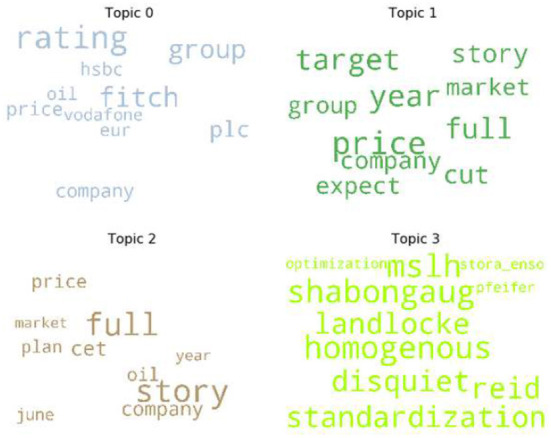
LDA topic modeling of our news stories corpus.

As each dataset contains slightly different topics and keywords, it would be interesting to assess whether a combination of three different datasets could help to improve the prediction of our model.

### 4.8. Results for topic modeling

We assessed whether topics derived from financial news and social media may provide accuracy in predicting market volatility. To do so, we built an LDA model to extract feature vectors from each day's news and then deployed logistic regression to predict the direction of market volatility the next day. To measure our classifier performance, we used the standard measures of accuracy, recall, precision, and F1 score. All these measures were obtained using the well-known Python Scikit-learn module^4^.

Table 2 summarizes the detailed results of our LDA and classification model. Again, all three models produced results which are in line with the previous studies of Atkins et al. (2018) and Mahajan et al. (2008). Despite the fact that the language used in tweets is informal, filled with acronyms and sometimes errors, the results we obtained from our Tweeter datasets were surprisingly good, with an accuracy that almost matches that obtained from the headlines dataset.

**Table 2:** Summary of the results.

**Dataset**	**Accuracy**	**Recall**	**Precision**	**F1 score**
Headlines	0.65	0.65	0.64	0.64
Tweets	0.64	0.65	0.70	0.64
Stories	0.67	0.67	0.81	0.63

## 5. Conclusions and future work

Our project aimed at performing correlation analysis to compare daily sentiment with daily changes in FTSE100 returns and volatility. To do so, we have created our own Web data extraction and database solution, orchestrating existing software and implementing all needed connectors.

Overall, our correlation analysis shows that sentiment captured from headlines could be used as a signal to predict market returns, but not so much volatility. The opposite was true for our tweets dataset. A correlation coefficient of –0.7, and *p*-value below 0.05 indicated that there is a strong negative correlation between positive sentiment captured from the tweets and the volatility of the market next day. It suggests that as the positive sentiment increases, market volatility decreases. Interestingly, the two variables move in the opposite direction, according to a vast literature in Empirical finance, in particular the so-called leverage effect observed both in the historical and implied volatility when the stock price and value of the firm go down.

Out of the three different data sets that were created, the most promising results came from the Headlines data set; this can be explained by the fact that this data set was the largest and had the longest time series. It would be beneficial to expand the correlation analysis by building a larger data corpus. In addition, we observed a slightly stronger correlation between sentiment captured from tweets containing cashtags ($) and market returns compared with tweets containing only hashtags (#) or multiple keywords. When it comes to building a tweet data set, there are some issues associated with hashtags or keywords. Many tweets will contain multiple keywords, but only actually express an emotion toward only one of them. Using more advanced natural language processing techniques to identify the subject of a tweet could potentially help reduce noise in Twitter data.

Results obtained with Granger's test indicate that, in general, sentiment obtained from news and social media does not seem to “cause” either changes in FTSE100 index prices or the volatility of the index; all *p*-values obtained in the tests where above 0.10 threshold so the null hypothesis could not be rejected. Yet the topics extracted from news sources can be used in predicting directional market volatility. It is interesting that topics alone contain a valuable information that can be used to predict the direction of market volatility. The evaluation of the classification model has demonstrated good prediction accuracy. Our model applied to the headlines dataset obtained an accuracy of 65%. It indicates that topics extracted from news could be used as a signal to predict the direction of market volatility the next day.

It was noticed that the accuracy of the model tends to depend on the number of topics chosen. There are different techniques that could be used to select an optimal number of topics; however, some of them, especially the development of high-frequency LDA models, are computationally expensive and would require a preliminary scalability analysis and a strong architecture to run on.

Future work on our architecture include building an *ad-hoc* sentiment scoring system or a system that detects mood (along the now-standard six axes of Calm, Alert, Sure, Vital, Kind, and Happy) from selected Financial news channels. In fact, previous work by Bollen et al. (2011) had indicated that the mood captured from tweets can help to predict the direction of Down Jones index with 86.7% accuracy. However, it would be interesting to see if this model could be improved by using a larger corpus of headlines and news stories, instead of tweets only. Finally, we want to emphasize the relatively small sample sizes used in this research and the interesting results we nevertheless obtained on our “low cost” infrastructure: a larger operation would give even better results.

## Data availability statement

The original contributions presented in the study are included in the article/supplementary material, further inquiries can be directed to the corresponding author.

## Author contributions

All authors listed have made a substantial, direct, and intellectual contribution to the work and approved it for publication.

## Footnotes

^1^Please see https://developers.refinitiv.com/eikon-apis/eikon-data-api.

^2^Please see the documentation on https://github.com/cjhutto/vaderSentiment.

^3^Please see https://github.com/marcmuon/nlp_yelp_review_unsupervised for an introduction to Kelechava's method.

^4^Please see https://scikit-learn.org/.
